# Micro-computed tomography assessment of different obturation techniques for filling lateral canals

**DOI:** 10.4317/jced.54806

**Published:** 2018-07-01

**Authors:** Mauro Fragachán, Montserrat Pons, Eduardo Barriuso, Jaime Frigola, Maria-Lluïsa Ballester, Esther Berástegui

**Affiliations:** 1 DDS. Master in Advanced and Experimental Endodontics. University of Barcelona, Spain; 2 DDS. Professor on Master’s Program in Advanced and Experimental Endodontics. University of Barcelona; 3 CORELAB, Senior Laboratory Technician, Marine Geosciences, Department of Earth and Ocean Dynamics, Faculty of Earth Sciences, University of Barcelona; 4 Associate Professor and CORELAB Laboratory manager, Marine Geosciences, Department of Earth and Ocean Dynamics, Faculty of Earth Sciences, University of Barcelona; 5 Graduate in Dental Medicine. Master in Endodontics. PhD. Researcher attached to the IDIBELL institute, Department of Endodontics, University of Barcelona. Professor on Master’s Program in Advanced and Experimental Endodontics. University of Barcelona; 6 Graduate in Dental Medicine. Master in Endodontics. PhD. MD. Researcher attached to the IDIBELL institute, Department of Endodontics, University of Barcelona. Director of the Master’s Program in Advanced and Experimental Endodontics, University of Barcelona

## Abstract

**Background:**

The aim of this study was to evaluate obturation depth and volume by means of micro-CT when filling lateral canals.

**Material and Methods:**

Thirty single-rooted teeth were used. After instrumentation, three artificial lateral canals were created on each mesial and distal surface (one on each third). The samples were then separated randomly into three groups according to the obturation technique used (n=10): lateral condensation (Group 1), Tagger’s hybrid technique (Group 2) and GuttaCore™ (Group 3). Samples were scanned and data was processed with Avizo software. Depth and volume of the infilling were measured in each lateral canal. Values were expressed as percentages and were analyzed using the Kruskal-Wallis test.

**Results:**

Mean depth showed statistically significant differences between Group 3 and Groups 1 (*p*=0.001) and 2 (*p*=0.003), whereas no significant difference was found between Groups 1 and 2 (*p*≈1). As for mean volume, significant differences were found between Group 3 and Groups 1 (*p*=0.01) and 2 (*p*=0.003) but no differences were found between Groups 1 and 2 (*p*=1.00).

**Conclusions:**

GuttaCore provided the best lateral canal sealing among the three techniques compared.

** Key words:**Lateral canals, Micro-CT, obturation.

## Introduction

According to several studies, the presence of lateral canals has been reported in 45 % of 74 teeth, 27.4 % of 1.140 teeth (1) and 75% of 493 (2) teeth. This represents an important finding that should be considered when performing endodontic treatment. Even though Ricucci & Siqueira stated that the belief that lateral canals must be injected with filling material to enhance treatment outcome was not supported by literature review or by their histopathologic observations (2), an adequate filling of lateral canals has been correlated with apical periodontitis healing and successful root canal treatment (3-5).

Different filling techniques have been proposed for achieving three-dimensional obturation of the root canal system. Classically, vertical compaction of warm gutta-percha, as proposed by Schilder, has obtained the best results for filling lateral canals (1,6). Tagger’s hybrid technique is a modification of a technique previously proposed by McSpadden (7,8). More recently, Guttacore (Dentsply Tulsa Specialties, Tulsa, USA) has been proposed as an improvement to the Thermafill system (Dentsply, Maillefer, Ballaigues, Switzerland). According to the manufacturer, it is similar to Thermafill with the difference that the gutta-percha carrier is made of interlaced gutta-percha that makes it possible to carry the previously heated gutta-percha through the canal to the apex providing three-dimensional filling.

Various experimental methods have been used to study the obturation quality of the root canal system. In endodontics, micro-computed tomography (micro-CT) has been used to evaluate root canal anatomy and morphology after biomechanical instrumentation (9). The method is highly accurate and nondestructive (10). A number of studies have evaluated the quality of root canal filling procedures by means of micro-CT (11-13) but as far as we are aware, none have evaluated lateral canal filling.

The purpose of this study was to evaluate, using micro-CT, the volume and depth of filling in artificial lateral canals comparing three obturation techniques: lateral condensation (LC), Tagger’s hybrid technique (TG), and GuttaCore (CG). The null hypothesis was that no significant differences would be found between GuttaCore and Tagger’s hybrid technique but differences would be found between Guttacore/Tagger’s hybrid and lateral condensation.

## Material and Methods

Thirty extracted and anonymized human single-rooted teeth were used in this study. The teeth were obtained from the C.0003113 collection (entered in the national register of biobanks, Carlos III Institute in Madrid, Spain). Teeth with calcified canals and curved roots were excluded. The study was approved by the Ethics Committee of the Bellvitge Dental Hospital of the University of Barcelona (Spain). Crowns were eliminated at the cementum-enamel junction using a low-speed hand-piece (NSK, Tokyo, Japan) and a 5113 HP diamond disk (Edenta, St. Gallen, Switzerland). The canal length was visually established by placing a K.10 file (Dentsply Maillefer, Ballaigues, Switzerland) until the tip was visible at the apical foramen. The working length was then established 1 mm short of the apex. Instrumentation was performed by using the X-Smart endo motor with Pathfile and ProTaper Next (Dentsply Maillefer) following the manufacturer’s protocol. All canals were instrumented up to X3 using 3 ml of 2.5% sodium hypochlorite for irrigation between each file. Afterwards, three artificial lateral canals were created on each mesial and distal surface of the root (one on each third) using an FG 858-014 EF high-speed extra-fine spear diamond bur (Coltene Whaledent, Langenau, Germany). Before obturation, a final flush with 5 ml of 17% EDTA and 5 ml of 2.5% sodium hypochlorite was performed and the canals were dried using paper points (Dentsply Maillefer). Teeth were randomly divided into 3 groups of 10 each. Two equally wide- experienced operators were randomly assigned five samples of each group. For all groups, AH Plus (Dentsply Maillefer) was used as canal sealer. The sealer was mixed following the manufacturer’s indications and, afterwards, carried to the canal using a gutta-percha point. The three obturation procedures were as follows.

Group 1 (LC): A gutta-percha point #30 was used as a master cone. Lateral condensation was performed using a B finger spreader (Dentsply Maillefer) and X-Fine (Dentsply Maillefer) gutta-percha accessory points. Excess gutta-percha was removed using a heated Mortonson #2 (Hu-Friedy, Chicago, USA) and compacted vertically with a Machtou plugger (Dentsply Maillefer).

Group 2 (TG): The procedure was similar to Group 1 except that the gutta-percha cones were thermomechanically compacted with a #50 Gutta-Condenser in rotation at 10.000 rpm with a low-speed hand-piece (NSK, Tokyo, Japan). Excess gutta-percha was managed in the same way as Group 1.

Group 3 (GC): A size verifier # 30 was applied as a first step. Once the sealer was applied as previously described, a #30 GuttaCore carrier, measured to working length, was placed in the Guttacore oven for 15 seconds and then applied to the canal with light pressure towards the apex until it reached the working length. The pressure was maintained for 10 seconds and then the carrier was cut manually as indicated by the manufacturer. Excess GuttaCore was managed in the same way as in Groups 1 and 2.

-Micro-CT scanning

To obtain high-resolution 3D densitometry maps of the inner structures of the teeth treated with the different substances in order to evaluate the infilling obtained, all samples were scanned by two double-blinded operators at the University of Barcelona CORELAB laboratory with a micro-computerized tomography (micro-CT) system, the MultiTom Core system supplied by X-ray Engineering (XRE), a spin-off company at the University of Ghent. This is a very versatile micro-CT system specially designed for long samples (up to 1.5 m long) that makes it possible to perform standard high-resolution micro-CT imaging on a wide range of objects and with a wide range of resolutions, from 300 to 5 µm. Treated tooth samples were scanned at tube conditions of 70kV and 32W using an Al filter, for a total of 1000 projections and an exposure time of 200 ms, resulting in a mean scan duration of 5 minutes, obtaining a voxel size resolution of 30 µm.

**Figure 1:**
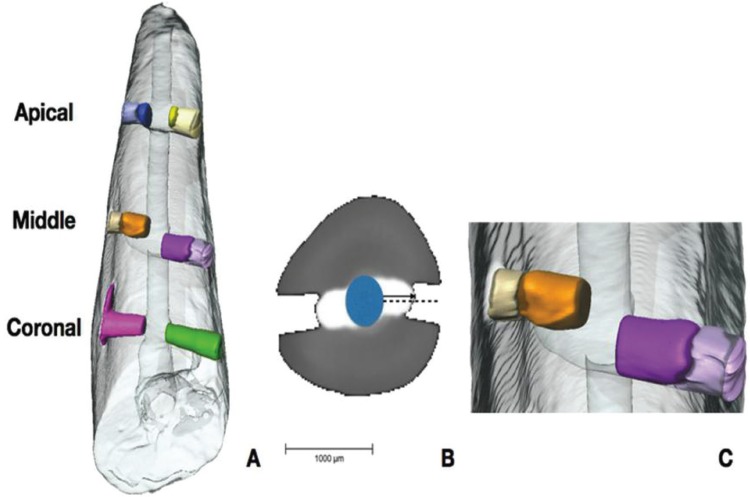
A) Volumetric 3D reconstruction of a sample in which materials have been segmented according to their density using AVIZO software: Tooth (transparent); obturation material (blue. yellow. orange. pink. purple and green for each lateral canal); and air (same color as canals but slightly lighter). B) Micro-CT slice to showing material of different densities on grayscale. White is the least dense and represents the obturation material; gray is the densest and represents the tooth. The dotted line represents the depth of the canal. whereas the continuous line represents the maximum distance reached by the obturation material. C) Detail of two lateral canals in the volumetric reconstruction shown in Figure A.

Data from each sample were reconstructed in 3D and filtered with RECON software (supplied by XRE) and, finally, segmentation of materials, depending on their density, was performed, i.e. tooth vs. infilling material and air (Fig. 1 A), using Avizo software. This is specialized software for 3D inspection and analysis of materials that makes it possible to obtain a huge amount of numerical data. For the purposes of this study the following measurements were taken: the maximum distance reached (depth) by infilling within the artificial orifice (Fig. 1 B); and the volume of each infilling (Fig. 1 C). Both were expressed as the percentages of the total length and volume of each orifice.

-Statistical analysis

Statistical analysis was performed applying the Kruskal-Wallis (K-W) nonparametric test. The level of significance was set at (*p*=0.05). When statistically significant differences were detected, a comparison between each pair of groups was performed. Mean values for each third (apical, middle, coronal) were calculated, as well as mean values for the whole sample.

## Results

-Depth

All depth percentages are summarized in  Table 1.

**Table 1:**
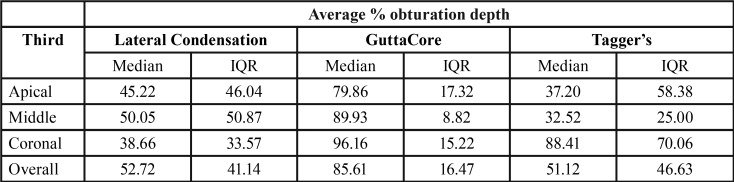
Percentage average obturation depth in different thirds of the root canal.

In analysis of the apical third, significant differences were not found between the 3 groups (K-W=5.26, *p*=0.072). However, in middle and coronal thirds significant differences were found. For the middle third, there were differences between the 3 groups (K-W=16.34, *p*<0.001). Pairwise comparisons found statistically significant differences between GuttaCore (GC) and Tagger’s (TG) (*p*<0.001), and between GC and lateral condensation (LC) (*p*=0.023), but there were no significant differences between TG and LC (*p*=0.59). Lastly, in the coronal third, significant differences were, again detected between the 3 groups (K-W=8.47, *p*=0.015). In this case, GC showed significant differences in comparison with LC (*p*=0.011), but no significant differences were found GC and TG (*p*=0.67) or LC and TG (*p*=0.28).

**Figure 2:**
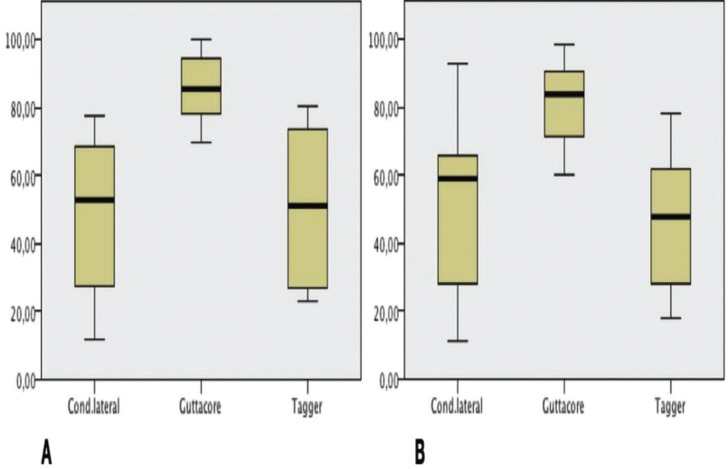
A) Obturation depth percentage. B) Obturation volume percentage.

Analyzing overall mean values, significant differences were found between the three groups (K-W=15.41, *p*=<0.001). Pairwise comparisons found that GC showed significant differences in comparison with LC (*p*=0.001), and TG (*p*=0.003), but no differences were found between LC and TG (*p*≈1) (Fig. 2).

Obturation depth results showed that GC achieved greater depth than TG and LC. In the middle third, GC was significantly more effective than TG and LC and, in the coronal third, GC depth was significantly greater than LC. These findings suggest that GC is more predictable and reliable than TG and LC as shown in the Interquartile range (IQR) ( Table 1).

-Volume

Volume percentages are summarized in  Table 2.

**Table 2:**
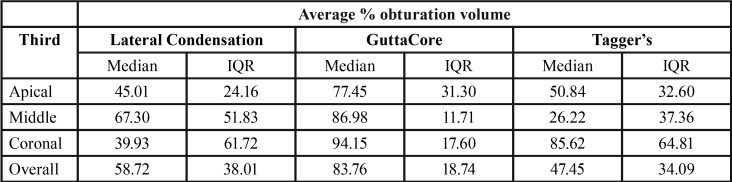
Percentage average obturation volume in different thirds of the root canal.

Analysis of the apical third found significant differences between the three groups (K-W=7.47, *p*=0.024). Pairwise comparisons found significant differences between GC and TG (*p*=0.043) but no differences were not between GC and LC (*p*=0.069); or TG and LC (*p*=0.99). For the middle third, significant differences were identified between the three groups (K-W=14.84, *p*=0.001). Pairwise comparisons found significant differences between GC and TG (*p*=0.001) but not between GC and LC (*p*=0.12) or TG and LC (*p*=0.22). Finally, in the coronal third, significant differences were found between the 3 groups (K-W=9.03, *p*=0.011). Unlike the previous thirds analyzed, GC showed statistically different values from LC (*p*=0.008), but no significant differences were found between GC and TG (p=0.34) or LC and TG (*p*=0.46).

**Figure 3:**
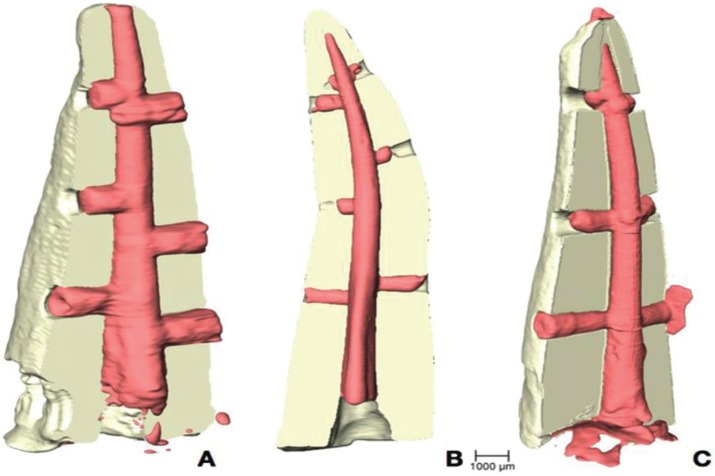
Volumetric 3D reconstruction in which the tooth and obturation material have been segmented in pink and beige. A) Group 1 sample: lateral condensation technique; B) Group 2 sample: Tagger’s hybrid technique; C) Group 3 sample: GuttaCore.

Analyzing overall mean values, significant differences were found between the three groups (K-W=13.14, *p*=0.001). Pairwise comparisons showed significant differences between GC and LC (*p*=0.01) and between GC and TG (*p*=0.003), but no differences were found between LC and TG (*p*=1.00) (Fig. 3).

For obturation volume, the present results also showed that GC obtained greater volume than TG and LC. Significant differences were found in both the apical and middle thirds in favor of GC, between GC and TG and between GC and LC in the coronal third ( Table 2).

## Discussion

Ramifications of the main canal, such as lateral canals, have great clinical importance in endodontic therapy especially when they are associated with lateral lesions (14).

Some authors have used resin blocks to study different obturation techniques for filling artificial lateral canals (15-17), whereas others have used natural teeth (1,14,18-22). In the present study, artificial lateral canals were created in natural teeth. The diameter was over 150 µm, as reported in previous investigations (23,24).

Micro-CT was used to evaluate lateral canal obturation volume and depth. Previous studies have used a variety of techniques to evaluate obturation: x-ray analysis (1,18), mono-dimensional observation through epoxy resin blocks (15), diaphanization (25) cross-sectioning (14,26) or longitudinal (27) observation. But micro-CT makes it possible to evaluate obturation homogeneity by obtaining three-dimensional images without sectioning or manipulating the samples, whereas all the other techniques described previously are based on two-dimensional images.

The present study evaluated lateral canal obturation depth and volume comparing three different filling techniques. Schäfer *et al.* (26) provided an evaluation method based on cross-sectional observation, that found better lateral canal filling outcomes with GC than LC. Clinton and Van Himel (27) obtained similar results using Thermafill rather than GC, using longitudinal sectional observation. But in the present study, no significant differences were found in obturation depth between TG and LC. The similarity in results could be explained by the possibility that when cold gutta-percha is compacted against the canal walls, the material deforms slightly (28), and only the sealer is squeezed into the lateral canals (20). Even so, Goldberg *et al.* (1), by means of radiographic evaluation, and Carvalho-Sousa *et al.* (14), assessing the obturation by cross-sectional observation, reported significant differences between TG and LC. These authors used similar techniques to the ones used in the present study with the difference that in the present study GuttaCore replaced continuous wave technique. The present study’s findings concur with Weine (3) and Wolcott *et al.* (29), who concluded that the obturation technique employed does not have an important effect on lateral canal filling.

As far as we are aware, no other studies assessing obturation volume by means of micro-CT have been published.

The better results obtained with GuttaCore, in terms of both depth and volume could be related to gutta-percha’s fluidity, which, when heated, facilitates its and sealer’s diffusion so that it will fill the isthmus, lateral, and accessory canals providing three-dimensional filling; it is also a simple technique to perform. Although several studies have proposed other obturation techniques for optimal lateral canal filling (4,6,15,17), others have proved that the use of thermoplasticized gutta-percha obtains better filling (1,14,15,19).

In the present study, the diameter of the lateral canals was about 700 µm, which is significantly larger than diameters reported in previous studies (150-200 µm) (23,24). Possible relations between the behavior of obturation materials and variations in lateral canal diameter constitute a subject for further research.

## Conclusions

The use of micro-CT to investigate lateral canal obturation by means of three-dimensional imaging and high and accurate resolution has made it possible to calculate obturation volume in lateral canal filling. This meant that it was possible to evaluate the quality of the obturation numerically. Based on the data obtained, GuttaCore showed better results in terms of both obturation depth and volume than Tagger’s hybrid technique and lateral condensation.
